# A phase I clinical trial of avelumab in combination with decitabine as first line treatment of unfit patients with acute myeloid leukemia

**DOI:** 10.1002/ajh.26043

**Published:** 2020-11-23

**Authors:** Hong Zheng, Shin Mineishi, David Claxton, Junjia Zhu, Chenchen Zhao, Bei Jia, W. Christopher Ehmann, Witold B. Rybka, Seema Naik, Natthapol Songdej, Joseph J. Drabick, Raymond J. Hohl

**Affiliations:** ^1^ Penn State Cancer Institute Penn State University College of Medicine Hershey Pennsylvania USA; ^2^ Department of Public Health Sciences Penn State University College of Medicine Hershey Pennsylvania USA

**Keywords:** AML, avelumab, decitabine, PD‐1, T cell exhaustion

To the Editor:

Despite considerable efforts, treatment of acute myeloid leukemia (AML) remains challenging. Prognosis for elderly patients or patients who are unfit for intensive chemotherapy is particularly poor as treatment options for them are very limited. Recent success using reagents targeting immune checkpoints, such as PD‐1, offers great promise for effective cancer therapy.^1^, ^2^ Several agents blocking the PD‐1 pathway have been FDA approved for treating multiple solid tumors and Hodgkin lymphoma. It has been demonstrated that hypomethylating agent (HMA) enhances the PD‐1 pathway in MDS and AML patients,^3^, ^4^ providing a strong rationale for combining HMA and PD‐1 inhibition in AML treatment. Avelumab is a PD‐L1 antibody that has been FDA approved for treating Merkel cell carcinoma, renal cell carcinoma, and urothelial carcinoma. Decitabine is a HMA that is commonly used in physicians' practice for treating AML patients who are unfit for intensive chemotherapy.

We performed a single arm, open label phase I study to evaluate safety and tolerability of avelumab in combination with decitabine in patients with untreated AML, who are unfit for intensive chemotherapy (NCT03395873). The trial was approved by the Institutional Review Board of Penn State University College of Medicine (STUDY7889). Written informed consent was obtained from all patients before enrollment. An initial stage (3 + 3 design) followed by an expansion stage of nine additional patients were designed. Patients in the initial stage cohort were monitored for dose‐limiting toxicity (DLT). The observation period for a DLT was a minimum of 28 days post induction therapy. The primary objective was to determine the safety of combinational treatment. Secondary objectives were to evaluate the complete remission (CR) rate and the overall survival (OS). Detailed information of patient selection, study design, treatment, and safety and response assessment is provided in Appendix S1.

Patient enrollment started January 2018, seven patients were enrolled by December 2018, at which time the accruement was discontinued (per the recommendation of Penn State University College of Medicine data and safety monitoring committee [DSMC]) for the best interest of patients due to the newly FDA approval of venetoclax, a novel treatment for the same patient population. However, all enrolled patients in this study continued treatment and a follow‐up was performed as per protocol defined. Table S1 summarizes the patients' characteristics. The median age was 71 years. Most patients (86%) carried adverse cytogenetics. All seven patients received at least one dose of avelumab and were included in the assessment of safety and survival. Two patients died of sepsis before response assessment by bone marrow biopsy, therefore five patients were evaluable for response.

No DLT was observed in the patient cohort of the initial stage. Two patients experienced grade three pneumonitis that was considered to be related to avelumab. One was in the initial cohort and the pneumonitis developed after the second cycle of treatment (beyond DLT evaluation period). The other was in the extension cohort. In both cases, pneumonitis resolved upon steroid treatment. However subsequent avelumab treatments were discontinued per protocol. The AEs were evaluated in all seven patients, Table S2 lists the nonhematologic AEs observed in more than one patient (>14%). The most common grade three or grade four AEs were febrile neutropenia (86%), hypoxia (57%), heart failure (29%), and pneumonitis (29%). Two patients died within 60 days after starting treatment. Both were due to sepsis, of which cellulitis was the infection source for one patient and dental abscess for the other.

Among the five patients who were evaluable for response, one patient (20%) achieved CR, one (20%) experienced progression of disease (PD), and three patients (60%) were with stable disease (SD) as the best response during the treatment course and follow up. All seven patients were assessed for survival. With a median follow‐up of 23.1 months, the median overall survival (OS) was 3.2 months (95% confident interval [CI], 1.2‐NR).

Comprehensive correlative studies were performed using blood samples collected from each patient. To investigate the effect of avelumab on immune response, we conducted complex flow cytometry‐based immune assays on samples prior vs 1 month post treatment. We observed no alteration in the frequency of each immune component (NK, NKT, B cells, DCs, monocytes, CD4 T cells, CD8 T cells, and Treg) upon avelumab and decitabine treatment (Figure 1A). When T cell differentiation subsets were examined based on the surface expression of CD45RA and CCR7, we found a significant increase in effector memory CD8 T cells. There was a trend of decreased terminal differentiated subsets, although no statistical significance was achieved likely due to limited sample size (Figure 1B). We next performed phenotypic and functional analysis of CD8 T cells. We observed a strong trend of up‐regulation of activation markers and co‐stimulatory receptors (CD69, CD226, CD38, ICOS and 4‐1BB) on CD8 T cells post treatment of avelumab and decitabine. In contrast, the expression of inhibitory molecules, including TIGIT, TIM‐3, CD160, LAG‐3, 2B4 and BTLA, were lower. Consistently, CD8 T cell function was enhanced in majority of patients, manifested by higher expression of granzyme B, perforin and Ki67, as well as more cytokine release (IFN‐γ and TNF‐α) upon in vitro TCR engagement (Figure 1C,D). Importantly, avelumab is likely the major contributor for the positive regulatory effect as studies on samples from patients who received decitabine alone did not show the same trend (Figure S1).

**FIGURE 1:**
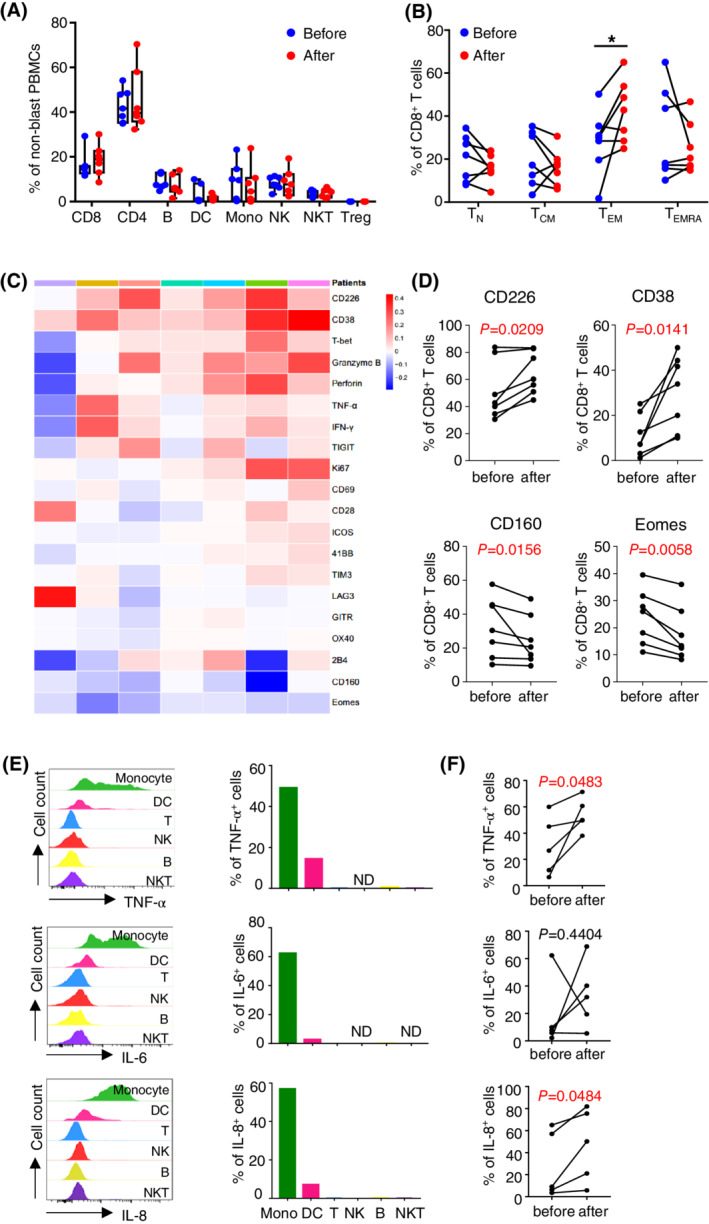
Effect of combination treatment on T cell response and inflammatory cytokine production in AML patients. (A‐D) Flow cytometry analyses of PBMCs collected from patients before and 28 days after combination treatment. A, Representative tSNE presentation (left) and plots (right) display the frequencies of each immune component. B, plots of CD8 T cell differentiation subsets, *p<.05. C, Heat map of immune markers that were normalized to a mean of 0 and S.D. of 1. Relative increase and decrease are assigned here as red and blue color, respectively. Each column represents one patient sample and each row represents an immune marker that was examined. D, Significant alterations of CD226, CD38, CD160, and Eomes before vs after treatment. E,PBMCs from a healthy donor were co‐cultured with LPS. Intracellular production of cytokines among each immune components was assessed by flow cytometry. (F) The PBMCs collected before and after combination treatment were co‐cultured with LPS. Cytokine production by monocytes were evaluated by flow cytometry. Plot summary (n = 5) is shown. ND, not detected

We observed grade three pneumonitis in two patients. In addition, five patients died of severe septic shock quickly after infections developed. Although neutropenic sepsis is common in AML patients under chemotherapy, the high severity of the inflammatory response is unusual. We hypothesize that avelumab‐mediated immune activity may contribute to the severity of patients' reaction. Proinflammatory cytokines are important regulators for the immune response during sepsis. To evaluate the impact of avelumab on the cytokine production by immune cells in response to infections, we performed ex vivo studies co‐culturing PBMCs with lipopolysaccharide (LPS), a major component of the outer membrane in Gram‐negative bacteria. Intracellular production of cytokines including TNF‐α, IL‐6, and IL‐8 was assessed by flow cytometry. Among all the immune components tested, we observed that cytokines were predominantly produced by monocytes upon LPS stimulation (Figure 1E). We then examined cytokine production by monocytes from samples collected post vs prior to the combination treatment. We found that upon ex vivo LPS stimulation, monocytes from most patients post avelumab and decitabine treatment had increased cytokine production compared to that of prior to treatment. Statistical significance was achieved in TNF‐α and IL‐8 (Figure 1F). We conducted the same study in samples from patients received decitabine alone treatment and found no impact of decitabine on the cytokine release (Figure S2). These data suggest that avelumab may increase the proinflammatory cytokine production during sepsis.

Comparing with historical data in AML patients treated with decitabine alone,^5^ we didn't observe an optimal clinical outcome in our patient cohort. Of note, the majority of patients (86%) enrolled in our study had AML with adverse risk per cytogenetic stratification, two patients (29%) had TP53 mutation and three with complex karyotype, both of which are considered very poor prognostic features. Our data is consistent with the results from the phase II study of Zeidan et al that azacitidine combined with durvalumab failed to show clinical benefit as the front‐line treatment for unfit AML patients.^6^ These observations highlight the need of optimal designs of clinical studies targeting PD‐1 for AML treatment. For instance, appropriate dose and timing of PD‐1 agents, as well as defining predictive biomarkers, are essential to improve clinical outcome for the combination treatment.

Inhibition of the PD‐1 pathway can effectively treat multiple cancers mainly through reversing T cell exhaustion and improving anti‐tumor T cell response. Consistently, we observed a positive impact of avelumab on T cell immune response in our cohort of AML patients. However, this improvement of T cell response didn't translate to a better clinical outcome. We made important findings that monocytes from patients treated with avelumab produce more proinflammatory cytokines upon ex vivo LPS stimulation. We suspect that avelumab‐caused high inflammatory response may contribute to the early death of the five patients who suffered neutropenic sepsis. In contrast to patients with solid tumors, whose blood counts including neutrophils are largely normal, AML patients frequently suffer infections due to persistent neutropenia. Infection‐triggered inflammatory response may turn severe in the presence of PD‐1Inhibition. This may explain why blockade antibodies to the PD‐1 pathway are successful in treating multiple solid tumors but their benefit to AML patients is limited.

In summary, although DLT was not detected in this phase I study, no clinical benefit was achieved in AML patients receiving avelumab and decitabine as first‐line treatment. In contrast, significant sepsis‐related death was observed. These data argue against the combination treatment at current design. Our correlative studies demonstrate that CD8 T cell response trends up upon avelumab treatment. However, the increased proinflammatory cytokines production during infections may exacerbate severe septic shock. Further mechanistic studies for better controlling the profound inflammation while maintaining anti‐leukemia T cell activity are essential to optimize PD‐1‐trageting treatment for AML.

## CONFLICT OF INTEREST

Dr. Hong Zheng received research funds from Pfizer for conducting the correlative studies of this trial.

## FUNDING INFORMATION

This work was supported in part by Penn State Cancer Institute (PSCI) Funds, the Penn State University Enhancing Health Initiative, the Kiesendahl Endowment funding, and an anonymous donation to PSCI for Cancer Research. The clinical trial was funded by Pfizer, as part of an alliance between Pfizer and Merck KGaA, Darmstadt, Germany.

## Supporting information


**Figure S1** The changes of CD8^+^ T cell immune markers from the AML patients who received decitabine treatment alone. Flow cytometry analysis was performed on the PBMC samples collected from 11 patients prior and 1‐month post to decitabine treatment. The percentages of positive cells according to various immune markers were gated and calculated. For the detection of TNF‐α and IFN‐γ cytokines, cells were stimulated with anti‐human CD3/CD28 antibodies (2 and 5 μg/mL; Ebioscience) supplemented with GolgiPlug (BD Biosciences) for 5 hours and performed intracellular staining followed by flow cytometry. The heat map shows the difference of post and prior treatment (post subtract prior) among immune markers on CD8^+^ T cells. The increase (results above zero) and decrease (results below zero) of the percentage of positive cells are assigned here as read and blue color, respectively. Each column represents one patient sample and each row represents an immune marker examined.

**Appendix** **S1** Supporting Information.

**Table S1** Patients' characteristics.
**Table S2.** Nonhematologic AEs in >14% of patients.

## Data Availability

The data that supports the findings of this study are available in the supplementary material of this article.
