# Supplementary figures and images for: A phase I clinical trial of avelumab in combination with decitabine as first line treatment of unfit patients with acute myeloid leukemia
Source: Am J Hematol. 2020 Nov 23;96(2):E46–50. doi: 10.1002/ajh.26043 (PMC7894154; doi:10.1002/ajh.26043)

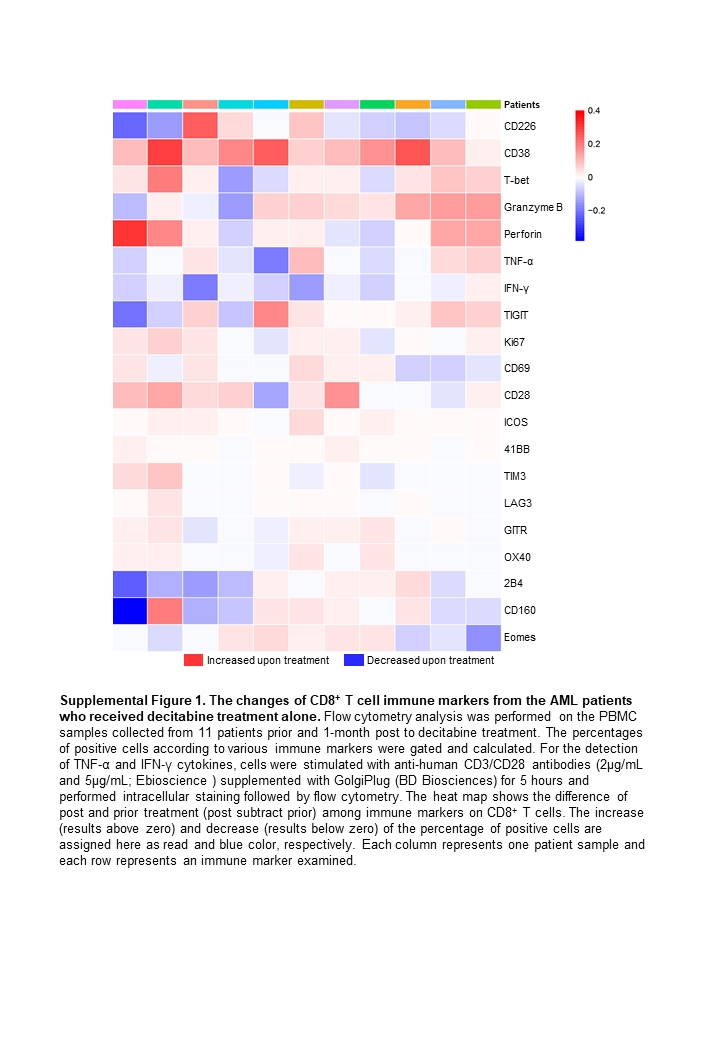

Supplement: Supplementary file 1 — Figure S1 The changes of CD8+ T cell immune markers from the AML patients who received decitabine treatment alone. Flow cytometry analysis was performed on the PBMC samples collected from 11 patients prior and 1‐month post to decitabine treatment. The percentages of positive cells according to various immune markers were gated and calculated. For the detection of TNF‐α and IFN‐γ cytokines, cells were stimulated with anti‐human CD3/CD28 antibodies (2 and 5 μg/mL; Ebioscience) supplemented with GolgiPlug (BD Biosciences) for 5 hours and performed intracellular staining followed by flow cytometry. The heat map shows the difference of post and prior treatment (post subtract prior) among immune markers on CD8+ T cells. The increase (results above zero) and decrease (results below zero) of the percentage of positive cells are assigned here as read and blue color, respectively. Each column represents one patient sample and each row represents an immune marker examined. [file AJH-96-E46-s001.tif]
