# Supplementary material for: A phase I clinical trial of avelumab in combination with decitabine as first line treatment of unfit patients with acute myeloid leukemia
Source: Am J Hematol. 2020 Nov 23;96(2):E46–50. doi: 10.1002/ajh.26043 (PMC7894154; doi:10.1002/ajh.26043)
Supplement: Supplementary file 2 — Appendix S1 Supporting Information. [file AJH-96-E46-s002.docx]

**Patients and Methods**

**Patient selection and study design**

This is a single arm, open label phase I study to evaluate safety and tolerability of avelumab in combination with decitabine in patients with untreated AML, who are unfit for intensive chemotherapy (NCT03395873). The trial was approved by the Institutional Review Board of Penn State University College of Medicine (STUDY7889). Written informed consent was obtained from all patients before enrollment. Adult patients with newly diagnosed AML (per World Health Organization 2008 criteria), except acute promyelocytic leukemia, were eligible if they are not considered as candidates for intensive remission induction chemotherapy based on ≥75 years of age or <75 years of age with at least 1 of the following: poor performance status (ECOG) score of 2; clinically significant heart or lung comorbidities; liver transaminases >3 × upper limit of normal; or other comorbidity the investigator judges incompatible with intensive remission induction chemotherapy. Patients who had prior AML-directed therapy, with the exception of hydroxyurea were not included. An initial stage (3+3 design) followed by an expansion stage (expansion cohort of 9 additional patients) were designed. Only one dose level was included in initial stage.

**Treatment regimen**

Decitabine was given 20mg/m2 IV day 1-5, every 28 days. Avelumab was given at 10mg/kg IV day 1, every 14 days. Treatment continues up to 1 year or until progression, unacceptable toxicity (ies), withdrawal of consent for any reason, lost to follow up, protocol violations, death for any cause.

**Safety assessment**

Patients in the initial stage cohort were monitored for dose-limiting toxicity (DLT). The observation period for a DLT was a minimum of 28 days post induction therapy. DLT was defined as any grade 3 or higher treatment-related non-hematologic toxicity, or any grade 4 neutropenia or thrombocytopenia that does not resolve to ≤ grade 3 within ≤ 7 days or ≤ grade 3 thrombocytopenia associated with any clinically important bleeding. Treatment-emergent adverse events (TEAEs) were assessed using the National Cancer Institute Common Terminology Criteria of Adverse Events (NCI-CTCAE) v4.03 (publication date: 14 June 2010). All TEAEs were noted, including any serious adverse events (SAEs), which were defined as AEs that resulted in death, were life-threatening, required inpatient hospitalization, resulted in significant disability or incapacity.

**Objectives and response criteria**

The primary objective was to determine whether avelumab in combination with decitabine is safe and tolerable for treatment of patients with AML. Secondary Objectives were to evaluate the complete remission (CR) rate and the overall survival (OS). Bone marrow aspirations and biopsies were performed post cycle 2 and 4 treatments to assess the response. CR was defined as bone marrow blasts < 5% with an absolute neutrophil count (ANC) > 1.0 × 109/L, platelet count > 100 × 109/L. CR with incomplete recovery (CRi) was defined as meeting all CR criteria except for residual neutropenia (< 1.0 × 109/L) or thrombocytopenia (< 100 × 109/L). Partial remission (PR) was defined as a decrease in pre-treatment bone marrow blast percentage by at least 50% and to within the range of 5–25%, while otherwise meeting all hematologic criteria of CR. Progressive disease (PD) was defined as >50% increase in marrow or peripheral blasts, or new extramedullary disease. Stable disease (SD) was defined as absence of CR, CRi, PR, and criteria for PD not met.

**Correlative studies**

To isolate PBMCs, peripheral blood samples were collected at indicated time from AML patients before or after the treatments. After 1:1 dilution of the samples with phosphate buffered saline (PBS), the peripheral mononuclear cells (PBMCs) were separated by centrifugation with Ficoll-Paque Plus solution (GE healthcare, Uppsala, Sweden). PBMCs were cryopreserved by slow programmable freezing in fetal bovine serum (FBS, Thermo Fisher Scientific, Waltham, MA, USA) supplemented with 10% dimethyl sulfoxide and stored in liquid nitrogen.

To perform flow cytometric analysis, PBMCs stored in liquid nitrogen were thawed and washed twice in PBS with 1% FBS (staining buffer). For cell surface staining, cells were first stained with Fixable Viability Dye eFluor 506 (eBioscience, San Diego, CA, USA) diluted in PBS for 20 min at 4℃. Then, Cells were washed with staining buffer twice and stained with fluorescence conjugated monoclonal antibodies (mAbs) for 20 min at 4℃. Transcription factor staining was performed with transcription factor buffer set (BD Parmingen, San Jose, CA, USA) after the cell surface staining. Permeabilized cells were then stained with mAbs for 30 min at 4℃. The samples were washed and resuspended in staining buffer or perm/wash buffer before flow cytometric analysis. LSR Fortessa flow cytometer (BD Biosciences, San Jose, CA) was used for data acquisition. Flow cytometry data was analyzed using FlowJo Software v10 (Tree Star, Ashland, OR, USA).

For *In vitro* stimulation and intracellular cytokine staining, PBMCs (~10^6^ cells/ml) were cultured in RPMI-1640 medium (Corning, Corning, NY, USA) supplemented with 10% FBS and stimulated with 100ng/ml LPS (MiliporeSigma, St.Louis, MO, USA) plus GolgiPlug (1μl/ml, BD Biosciences) in CO_2_ incubator. For TNF-α and IL-6 detection, cells were stimulated for 4 hours; for IL-8 detection, cells were stimulated for 6 hours. After *in vitro* stimulation, cells were washed and stained with Fixable Viability Dye eFluor 506 as described above. Then, cell surface staining was performed using fluorescence conjugated anti-human mAbs, CD45-BV786, CD3-APC, CD19-FITC, CD14-BV711, CD11b-AF700, CD33-APC-Cy7, CD56-PE-CF594 and HLA-DR-PE-Cy7 (BD Biosciences) for 20 min at 4℃. Cells were washed with staining buffer and fix/permeabilized with BD cytofix/cytoperm kit (BD Biosciences) following manufactural instructions. Intracellular staining was performed using anti-human TNF-BV421, IL-6-PE and IL-8-BV421 (BD Biosciences) for 30 min at 4℃. Data acquisition and analysis were same as described above.

**Statistics**

Descriptive statistics were used to summarize patients’ demographic information, baseline clinical characteristics, and main clinical outcomes. Kaplan-Meier curve was generated to illustrate patients’ overall survival time. Paired tests (either paired t-test or nonparametric Wilcoxon Signed-rank test for paired data) were used to compare the biomarker values before and after treatment. Most analyses were done using statistical software SAS version 9.4 (SAS Institute, Cary, NC, USA). The hierarchical clustering and heatmaps were obtained using heatmap package in R. GraphPad Prism (GraphPad Software Inc., San Diego, CA, USA) and R version 3.5.1 were used for statistical calculations and visualization. All tests were two-sided and the statistical significance level used was 0.05.
