# Supplementary material for: A phase I clinical trial of avelumab in combination with decitabine as first line treatment of unfit patients with acute myeloid leukemia
Source: Am J Hematol. 2020 Nov 23;96(2):E46–50. doi: 10.1002/ajh.26043 (PMC7894154; doi:10.1002/ajh.26043)
Supplement: Supplementary file 3 — Table S1 Patients' characteristics. Table S2. Nonhematologic AEs in >14% of patients. [file AJH-96-E46-s003.docx]

| Subject ID | Age/Sex | PS | Co-Morbidities | Cytogenetics/Risk Stratification | |
| --- | --- | --- | --- | --- | --- |
| 1 | 71/ M | 2 | COPD | Normal karyotype,  Mutation: Flt-3 ITD, EZH2, RUNX1, SF3B1 | Adverse |
| 2 | 71/ F | 1 | HTN | Del (5q)  Mutation: BCOR and PTPN11 | Adverse |
| 3 | 62/ M | 1 | COPD, DM, NSTEMI, AAA | Complex karyotype,  Mutation: TP53 and Kit | Adverse |
| 4 | 71/ M | 0 | CAD, PVD, HTN | Normal karyotype  Mutation: PTPN11, IDH2, RUNX1, BCORL1, NRAS, SRSF2, EZH2, SF3B1. | Adverse |
| 5 | 76/ M | 2 | HTN, CKD | Complex karyotype | Adverse |
| 6 | 78/ M | 0 | HTN, BPH | Complex karyotype  Mutation: TP53 | Adverse |
| 7 | 67/M | 1 | COPD | NPM-1, Flt-3 TKD, DNMT3A, NRAS | Favorable |

**Supplemental Table 1.** Patients’ characteristics

**Supplemental Table 2.** Nonhematologic AEs in >14% of patients

| **Adverse Event** | **Grade, Number** | | | | **Total (%)** |
| --- | --- | --- | --- | --- | --- |
|  | **1** | **2** | **3** | **4** |  |
| Fatigue | 2 | 4 | 1 | 0 | 7 (100) |
| Weight loss | 4 | 2 | 1 | 0 | 7 (100) |
| Febrile neutropenia | 0 | 0 | 5 | 1 | 6 (86) |
| Hypertension | 0 | 2 | 4 | 0 | 6 (86) |
| Anorexia | 3 | 3 | 0 | 0 | 6 (86) |
| Edema | 3 | 2 | 1 | 0 | 6 (86) |
| Pleural effusion | 4 | 2 | 0 | 0 | 6 (86) |
| Hypoxia | 0 | 1 | 3 | 1 | 5 (71) |
| Acute kidney injury | 2 | 2 | 1 | 0 | 5 (71) |
| Diarrhea | 3 | 2 | 0 | 0 | 5 (71) |
| Dizziness | 4 | 1 | 0 | 0 | 5 (71) |
| Hypotension | 3 | 2 | 0 | 0 | 5 (71) |
| Hypokalemia | 1 | 1 | 2 | 0 | 4 (57) |
| Oral mucositis | 2 | 1 | 1 | 0 | 4 (57) |
| Cough | 3 | 1 | 0 | 0 | 4 (57) |
| Headache | 3 | 1 | 0 | 0 | 4 (57) |
| Nausea | 4 | 0 | 0 | 0 | 4 (57) |
| Vomiting | 4 | 0 | 0 | 0 | 4 (57) |
| Urinary retention | 1 | 2 | 0 | 0 | 3 (43) |
| Pneumonitis | 0 | 0 | 2 | 0 | 2 (29) |
| Heart failure | 0 | 0 | 2 | 0 | 2 (29) |
| Atrial fibrillation | 2 | 0 | 0 | 0 | 2 (29) |
| QTc prolonged | 1 | 1 | 0 | 0 | 2 (29) |
| Abdominal pain | 2 | 0 | 0 | 0 | 2 (29) |
| Constipation | 1 | 1 | 0 | 0 | 2 (29) |
| Myalgia | 2 | 0 | 0 | 0 | 2 (29) |
| Rash | 1 | 1 | 0 | 0 | 2 (29) |
